# Rickets: Treatment by Phosphorus

**Published:** 1891-01-31

**Authors:** 


### The Practitioner's Mirror and Retrospect.

*[The Editor will be glad to receive offers of co-operation and contributions from members of the profession. There is no doubt that much valuable material full of interest and instruction is at present lost to science. This is largely so in the case of* (1) *the younger and less-known members of the hospital staff* (2) *those connected with cottage hospitals, and* (3) *the great body of medical practitioners not attached to any institution. The co-operation of all is cordially invited to enable the Editor to make* The Hospital *of the utmost possible use and value to the profession. Specialists willing to review books on their special subjects are invited to communicate this fact at once. All letters should be addressed to* The Editor, The Lodge, Porchester Square, London, W.]

### MEDICINE.

### RICKETS: TREATMENT BY PHOSPHORUS.

As far back as 1872 Wegner investigated experimentally the effects of phosphorus on the growth of bone, and appeared to demonstrate conclusively that phosphorus acts as a general stimulant to the structures concerned in the formation of bone. If phosphorus was given in comparatively large doses it stimulated these structures so violently as to produce necrosis. It was in this manner that phosphorus fumes gave rise to the "phosphorus necrosis," which was formerly so common in matchmakers. The very interesting fact that when administered to young and growing animals from which lime was withheld, there was an excessive production of osteoid tissue, very similar to that which is found in rickets, and that under like conditions Guérin observed the same effects, may account for the injurious results that Lorey, Henoch, and Monti have noticed from its administration in this disease, and which led them to affirm that phosphorus was contra-indicated in its treatment. We are further reminded of the very favourable experience of Kassowitz with phosphorus in rickets. In his experiments upon animals he found that the compact portion of bone was increased at the expense of the cancellous tissue, but he further ascertained that this increase was due to a shrinking of the medullary spaces, and not to a fresh deposition of bony plates. On increasing the dose little by little, however, he at last came to a point where the action of the drug appeared to be reversed.

Dr. Mandelstamm, of Kazan, Russia, has recently published his observations on 216 cases of rickets treated with small doses of phosphorus with most gratifying results. His patients were between the ages of three years and three months, the phosphorus being given in doses of about $\frac{1}{70}$th to $\frac{1}{15}$th of a grain a day, dissolved in almond oil. The children were treated for several months, and even, in some cases, for a year. Generally after two months of this treatment the bones of the skull became more compact, the fontanelles and sutures less prominent, and the nervous crises and paroxysms of laryngeal stridor less frequent and pronounced. The growth and weight of the body generally was also observed, and the measurements of the head and chest and it was found that the increase in weight and general improvement in health were likewise most obvious.

All the patients were of the poorer classes, and the author believed that, considering the adverse hygienic and dietetic conditions under which the treatment was necessarily carried out, the improvement could only be attributed to the phosphorus, no injurious effects arising from the use of the drug, but cases in which catarrh of the stomach and intestines exist should be first treated for this condition, before the treatment with phosphorus is commenced.

The treatment of rickets by phosphorus is not an innovation, but it has never been widely adopted in practice. The results of Dr. Mandelstamm are certainly most encouraging, and deserve careful consideration; but how are we to reconcile his results and those of Kassowitz and others, with the diametrically opposite results obtained by equally competent observers? It would seem that the essential point to be remembered in the use of phosphorus in this disease is that the doses be small, and that the phosphorus be in a free condition. It is useless to administer phosphoric acid or the phosphates under the impression that we are obtaining the medical effects of phosphorus. That lime salts are below the normal amount in rickety bones, even as low often as 21 per cent., is an established fact, but that rickets is due to a deficiency in the amount of lime in the food or in the amount assimilated is very far from certain. If this deficiency was the *sole* cause of the changes in the bones, we should expect to find the ossifying bones normal in all other respects. This, however, is not the case, for the histological appearances can only lead us to regard the pathological condition as essentially *developmental*. Rickets is a general affection, not local merely, and if the physiological action of phosphorus was merely local, it would be difficult to explain the general improvement in Dr. Mandelstamm's patients. The general hygienic treatment of rickets gives very favourable results, and undoubtedly acts indirectly, in inducing normal ossification locally. But it would seem that in phosphorus we have a drug which combines general improvement with a direct local action, and is, in fact, apparently a "specific" for rickets. Dr. Mandelstamm's mode of administration of phosphorus is worthy of note. Most of the pill masses of phosphorus are useless, being made with insoluble materials which pass through the bowel unchanged. It may be given in cod-liver oil or dissolved in olive oil, but it must be freshly prepared, as it is almost impossible to devise any liquid formula which does not soon become inert by oxidation.

